# Could elective cesarean sections influence the birth weight of full-term infants?

**DOI:** 10.1590/S1516-31802006000600002

**Published:** 2006-11-01

**Authors:** Eddie Fernando Candido Murta, Guilherme Carvalho Freire, Daniel Capucci Fabri, Renato Humberto Fabri

**Keywords:** Cesarean section, Parturition, Birth weight, Birthing centers, Health behavior, Cesárea, Parto, Peso ao nascer, Centros independentes de assistência à gravidez e ao parto, Conduta de saúde

## Abstract

**CONTEXT AND OBJECTIVE:**

There are no studies on birth weights among full-term infants born by means of elective cesarean section. We aimed to study this in private and public hospitals.

**DESIGN AND SETTING:**

Retrospective study at Universidade Federal do Triangulo Mineiro, Uberaba, Brazil.

**METHODS:**

Data were collected from the municipal medical birth register of Uberaba from January to December 2000. The data obtained (maternal age, type of delivery, number of prenatal care visits and birth weight, from full-term pregnancy) from the university hospital (UH), which is a tertiary hospital that only attends patients within the National Health System (SUS), were compared with data from four private hospitals (PHs) that attend health insurance plans and private patients. Student's t test, χ^2^ test and multiple logistic regression were used for statistical analysis, with the significance level set at p < 0.05.

**RESULTS:**

In the PHs, 1,100 out of 1,354 births (81.2%) were by cesarean section and in the UH, 373 out of 1,332 (28%). Birth weight increased significantly in association with increasing numbers of prenatal care visits, except for cesarean section cases in PHs. Birth weights among vaginal delivery cases in PHs were greater than in the UH (p < 0.05), but this was not observed among cesarean section cases. Multiple logistic regression showed that there was greater risk of low birth weight in PHs (odds ratio: 2.33; 95% confidence interval: 1.19 to 4.55).

**CONCLUSION:**

Elective cesarean section performed in PHs may be associated with low birth weight among full-term infants.

## RESUMO

**CONTEXTO E OBJETIVO:**

Uma das principais causas do aumento das taxas de cesariana no Brasil é o predominante caráter eletivo das indicações, principalmente em hospitais privados. O risco de desconforto respiratório neonatal pode ser uma complicação da cesariana eletiva, mas pelos nossos conhecimentos, não há estudo sobre peso ao nascimento de fetos a termo. Portanto, o objetivo deste trabalho é estudar o peso de fetos a termo nascidos em hospitais privados e públicos.

**TIPO DE ESTUDO E LOCAL:**

Estudo retrospectivo realizado de Janeiro a Dezembro de 2000 na Universidade Federal do Triângulo Mineiro, em que os dados foram coletados do registro médico de nascimento de Uberaba (Brasil).

**MÉTODOS:**

Nós comparamos os dados obtidos (idade materna, tipo de parto, número de consultas prénatais e o peso ao nascer de fetos a termo) no Hospital Universitário (HU), hospital terciário que atendopelo Sistema Único de Saúde (SUS) com quatro hospitais da rede privada (HPs – Hospitais Particulares) que atendem planos de saúde e privados. Os testes de "T de Student's", "χ^2^" e regressão logística múltipla foram usados para análise estatística com nível de significância estabelecido em p < 0,05.

**RESULTADOS:**

1.100 em 1.354 (81,2%) e 373 em 1.332 (28%) cesarianas ocorreram, respectivamente, no HPs e HU. Nós observamos que o peso ao nascimento aumenta significantemente em associação com o número de consulta pré-natais, com exceção das cesarianas no HPs. Além disso, o peso ao nascimento nos partos vaginais no HPs é maior que no HU (p < 0,05) mas isto não é observado nas cesarianas. A análise de regressão múltipla demonstrou que o risco de baixo peso ao nascer no HPs é 2,33 (1,19 to 4,55).

**CONCLUSÃO:**

As cesarianas eletivas realizadas nos hospitais privados podem estar associadas a um menor peso ao nascimento de fetos a termo.

## INTRODUCTION

Increasing cesarean section rates are a worldwide phenomenon, especially in Brazil, but they still cannot be fully explained.^1-3^ The culture of cesareans in Brazil affects obstetricians and society in general.^3^ One of the causes of the increasing cesarean section rates in Brazil is the predominantly elective nature of the indications, which are seen mainly in private hospitals.^4,5^ Elective cesarean section rates have also been increasing around the world.^5^

Neonatal respiratory distress risks may be a complication in elective cesarean sections.^6^ In Brazil, some studies have showed increased rates of preterm births and low birth weight (< 2500 g), probably due to iatrogenic practices related to elective cesarean sections.^7,8^ But to our knowledge, there have not been any studies on birth weights of full term infants born by means of elective cesarean section. Dystocia is more frequent in prolonged gestation, because of the increased fetal weight.^9^ Since birth weight increases as pregnancy progresses,^9^ the question that arises is whether elective cesarean section performed prior to spontaneous labor could influence the birth weight. In theory, infants born from elective cesarean section at a gestational age of 37 weeks will have lower birth weight than if they were born at a gestational age of 40 weeks.

## OBJECTIVE

We had the aim of studying birth weights among full-term infants born from cesarean section and vaginal delivery at private hospitals and public hospitals.

## METHODS

A retrospective study was conducted at Universidade Federal do Triângulo Mineiro, Uberaba, Brazil, from January to December 2000, in which data were collected from the municipal medical birth register of Uberaba. Maternal age, type of delivery, number of prenatal care visits and birth weights of newborns from full-term pregnancies (3741 weeks of gestation) were analyzed. The length of gestation was measured from the first day of the last menstrual period. Twin pregnancies were excluded.

We compared data obtained from the university hospital (UH) and four private hospitals (PHs). The UH is a tertiary hospital that only attends patients within the National Health System (SUS) who have an obstetric indication for cesarean section. This hospital provides free attendance for a population of low socioeconomic and educational levels. The PHs provide attendance for patients with health insurance plans and private patients and accept elective indication for cesarean section. These patients have higher socioeconomic and educational levels.

Student's t test, χ^2^ test and multiple logistic regression were used for statistical analysis, with the significance level set at p < 0.05. The Graph Pad Prism 3.0, Statistical Package for Social Sciences (SPSS) and Epi Info 6.04 software were used for data analysis. It was calculated that the sample size should be at least 250 (Student's t test with mean difference of 100 grams and anticipated standard deviation of 400 grams; χ^2^ test with difference of 20%; both with statistical power of 80%).

This research was approved by the Research Ethics Committee of Universidade Federal do Triângulo Mineiro.

## RESULTS

The type of medical attendance exerted an influence on the type of delivery and in birth weight among the expectant mothers (Table 1). In the PHs, 1,100 out of 1,354 births (81.2%) were by cesarean section, while in the UH 373 out of 1,332 births (28%) were cesareans. Birth weight increased significantly in association with increasing numbers of prenatal care visits, with the exception of cesarean section cases in PHs. Moreover, birth weights among vaginal delivery cases in the PHs were greater than in the UH (p < 0.05), but this was not observed among cesarean section cases.

**Table 1:** Distribution of birth weights (in grams, expressed as mean and standard deviation), maternal age (years, expressed as mean and standard deviation), and number of prenatal care visits during pregnancy (number and percentage of patients with more than six visits) according to hospital and type of delivery

	Deliveries in university hospital (UH)		Deliveries in private hospitals (PH)	
	Vaginal (n = 959)	Cesarean (n = 373)	Vaginal (n = 254)	Cesarean (n = 1,100)
Birth weight*	3,132 (429.6)	3,279 (481.7)	3,185 (429.9)	3,312 (420.3)
Maternal age	22.5 (5.4)	24.5 (6.3)	24.6 (5.9)	26.8 (6)
Prenatal care visits†	384 (40%)	203 (54.4%)	218 (85.8%)	1,060 (96.3%)

* *all comparisons were significant (p < 0.05, Student's t test), except for birth weight among cesarean section cases in PHs versus UH;*

† *all comparisons were significant (p < 0.0001, χ^2^ test).*

Multiple logistic regression showed that the risk of low birth weight among women undergoing cesarean section was greater in the PHs (adjusted odds ratio: 2.33; 95% confidence interval: 1.19 to 4.55) than for infants born by cesarean section in the UH. The reference standard utilized was vaginal delivery in the UH (Table 2).

**Table 2:** Results of multiple logistic regression comparing low birth weight (< 2500 g) at the university hospital and private hospitals, in relation to the variables analyzed, expressed as odds ratio (with 95% confidence interval)

Variables	University hospital	Private hospitals
Delivery		
Vaginal	1.0	-
Cesarean section	1.4 (0.82 to 2.4)	2.33 (1.19 to 4.55)*
Mother's age (years)		
≥ 20	1.0	10.3 (4.7 to 22.5)*
< 20	1.44 (0.53 to 3.9)	1.12 (0.61 to 2.07)
Prenatal care visits		
≥ 6	2.04 (0.85 to 4.9)	1.05 (0.26 to 3.7)
< 6	1.0	2.4 (1.18 to 4.88)*

* *p < 0.05.*

## DISCUSSION

The UH is a tertiary reference hospital that attends higher risk pregnant women than do the PHs. This helps to explain the statistical difference in birth weight between the vaginal delivery cases in the UH and those in the PHs. We had expected that the birth weights among vaginal delivery and cesarean section cases would be significantly higher in the PHs than in the HU, but this occurred only among the vaginal delivery cases.

Another noteworthy point was the high frequency of prenatal care visits among the cases attended in the PHs. Birth weight increased with increasing numbers of prenatal care visits, except for the birth weight in cesarean section cases, comparing the UH with the PHs. Multiple logistic regression analysis showed that, although there were greater numbers of prenatal care visits among the PH cases than among the UH cases, the risk of low birth weight among cesarean sections performed in the PHs was greater. For us, this is an intriguing finding. It reinforces our hypothesis that elective cesarean section may have an influence on birth weight.

Birth weight alterations have been demonstrated in cases of breech presentation and repeated cesarean section, and these may also support our hypothesis. The Term Breech Trial showed that women allocated to planned cesareans delivered earlier and had fewer large newborns than did those for whom a vaginal delivery was planned.^10^ Another study showed that 7.8% of the newborns had birth weights of more than 3000 g in cases of repeated cesarean section, versus 25.3% and 30.9%, respectively, for cases of vaginal delivery and first cesarean section.^11^ For these authors, this observation coincided with the main indications for the first cesarean section group: dystocia or cephalopelvic disproportion.^11^

Increased preterm delivery^12^ and low birth weight^9^ may be due to elective cesarean section. The increased trend towards low birth weight was restricted to the 36^th^ and 37^th^ weeks of gestation in one study,^9^ and to the period from 36 to 40 weeks in another.^8^ A study in northeastern Brazil^13^ showed that low birth weight was associa ted with low maternal height, maternal smoking, primi parity, previous low birth weight, use of public health service rather than private healthcare, preterm birth and cesarean section. These authors concluded that abusive indications for elective cesarean section could cause low birth weight. It is important to emphasize that our data were coded by time period (full term was defined as the period from 37 to 41 weeks of gestation), while it is likely that an infant born from elective cesarean section at a gestational age of 37 weeks will have lower birth weight than if it was born at a gestational age of 40 weeks.

We believe that further studies that control for multiple variables such as parity, multiple pregnancy, medical complications such as hypertension, and fetal complications such as intrauterine growth restriction, could add more information to our results. Whether birth weight alterations among full-term infants can increase newborn complications or affect child development is a complex question for which there are at present no answers. A multi-center study with multi-analysis of these variables could support our hypothesis.

## CONCLUSIONS

Taken together, our findings suggest that elective cesarean section performed in private hospitals may be associated with low birth weight among full-term infants.

## References

[1] Odlind V, Haglund B, Pakkanen M, Otterblad Olausson P (2003). Deliveries, mothers and newborn infants in Sweden, 1973-2000. Trends in obstetrics as reported to the Swedish Medical Birth Register. Acta Obstet Gynecol Scand.

[2] Wu WL (2000). Cesarean delivery in Shantou, China: a retrospective analysis of 1922 women. Birth.

[3] Nuttall C. (2000). Caesarean section controversy. The caesarean culture of Brazil. BMJ.

[4] Fabri RH, Murta EF (2002). Socioeconomic factors and cesarean section rates. Int J Gynaecol Obstet.

[5] Murta EF, Nomelini RS (2004). Is repeated caesarean section a consequence of elective caesarean section?. Lancet.

[6] Hannah ME (2004). Planned elective cesarean section: a reasonable choice for some women?. CMAJ.

[7] Silva AA, Barbieri MA, Gomes UA, Bettiol H (1998). Trends in low birth weight: a comparison of two birth cohorts separated by a 15-year interval in Ribeirão Preto, Brazil. Bull World Health Organ.

[8] Barbieri MA, Silva AA, Bettiol H, Gomes UA (2000). Risk factors for the increasing trend in low birth weight among live births born by vaginal delivery, Brazil. Rev Saude Publ.

[9] Turner MJ, Rasmussen MJ, Turner JE, Boylan PC, MacDonald D, Stronge JM (1990). The influence of birth weight on labor in nulliparas. Obstet Gynecol.

[10] Hannah ME, Hannah WJ, Hewson SA, Hodnett ED, Saigal S, Willan AR (2000). Planned caesarean section versus planned vaginal birth for breech presentation at term: a randomised multicentre trial. Term Breech Trial Collaborative Group. Lancet.

[11] Trujillo-Hernandez B, Rios-Silva M, Huerta M, Trujillo X, Vasquez C, Millan-Guerrero R (2002). Frequency of indications for and clinical epidemiological characteristics of first time cesarean section, compared with repeated cesarean section. Arch Gynecol Obstet.

[12] Bettiol H, Rona RJ, Chinn S, Goldani M, Barbieri MA (2000). Factors associated with preterm births in southeast Brazil: a comparison of two birth cohorts born 15 years apart. Paediatr Perinat Epidemiol.

[13] Silva AA, Lamy-Filho F, Alves MT, Coimbra LC, Bettiol H, Barbieri MA (2001). Risk factors for low birthweight in north-east Brazil: the role of caesarean section. Paediatr Perinat Epidemiol.

